# Seropositivity for *Coxiella burnetii* in suspected patients with dengue in São Paulo state, Brazil

**DOI:** 10.1371/journal.pntd.0010392

**Published:** 2022-05-10

**Authors:** Danilo Alves de França, Mateus de Souza Ribeiro Mioni, Felipe Fornazari, Ana Íris de Lima Duré, Marcos Vinicius Ferreira Silva, Fábio Sossai Possebon, Virgínia Bodelão Richini-Pereira, Helio Langoni, Jane Megid

**Affiliations:** 1 Department of Veterinary Hygiene and Public Health, Paulista State University “Júlio de Mesquita Filho”, Botucatu, São Paulo, Brazil; 2 Octávio Magalhães Institute, Ezequiel Dias Foundation, Belo Horizonte, Minas Gerais, Brazil; 3 Adolfo Lutz Institute, Regional Laboratories Center II, Bauru, São Paulo, Brazil; University of Texas Medical Branch at Galveston, UNITED STATES

## Abstract

Q fever and brucellosis are zoonoses that cause fever and other systemic clinical signs in humans; their occurrences are neglected and the differential diagnosis for some diseases is disregarded. This study aimed to investigate the seropositivity for *Coxiella burnetii* and *Brucella* spp. antibodies in patients suspected of dengue from 38 municipalities in the state of São Paulo, Brazil. The samples (n = 604) were obtained by convenience from the Adolfo Lutz Institute serum bank. Sera were subjected to an indirect immunofluorescence assay (IFA) using *in-house* and commercial diagnostic protocols to evaluate *C*. *burnetii* positivity. For *Brucella* spp., sera were subjected to rapid plate serum agglutination with buffered acidified antigen (AAT), slow tube serum agglutination (SAL), and 2-mercaptoethanol (2-ME) techniques. Associations and statistical inferences of the results were performed by logistic regression according to the clinical and demographic variables collected from the patients. Statistical analyses were performed using Statistical Analysis Software (SAS) and associations were considered when p value was <0.05. In all, 129 patients showed positive results for Q fever, indicating a seropositivity of 21.4% (95% CI 18.15–24.85). Patients with 14–20 days of symptoms had 2.12 (95% CI 1.34–3.35) times more chances of being seropositive for Q fever than patients with 7–13 days, and patients with 21–27 days of fever had 2.62 (95% CI 1.27–5.41) times more chances of being seropositive for Q fever than patients with 7–13 days. For the other variables analyzed, there were no significant associations between the groups. No positivity for brucellosis was observed. This is the most comprehensive study of people seropositive for Q fever in São Paulo state and provides additional data for the medical community in Brazil. It is suggested that Q fever may be an important differential diagnosis of febrile illnesses in the region, demanding the government’s attention and investment in health.

## Author summary

Brazil is a tropical country with a warm climate and large animal herds that favor many zoonotic agents that circulate in the territory. Due to the high prevalence of some tropical diseases, such as dengue, malaria, and chikungunya, important zoonoses have been neglected during the clinical evaluation of patients, resulting in misdiagnosis. Examples include Q fever and brucellosis, zoonoses that occur in animals in Brazil and are easily transmitted to humans through contaminated air and food products. There is not much data on these diseases in humans in the country representing their actual health situation. The current knowledge is supported by a few serological surveys developed by research groups and case reports. In this study, sera from patients with fever of unknown origin were positive for Q fever and brucellosis. The authors found that, contrary to brucellosis, Q fever has affected many people in São Paulo, the most populous state in the country. In addition, other findings regarding patient profile and disease behavior have been established. This research highlights the need for surveillance and control of Q fever in humans and animals to prevent acute cases and treat chronic patients.

## Introduction

*Coxiella burnetii* is the causative agent of Q fever, a zoonosis of easy transmissibility and underestimated in Brazil [1]. It is a neglected disease in the country with an unknown prevalence. The high percentage of asymptomatic individuals (near 60%) and the unspecific acute symptoms, characterized as flu-like, contribute to the lack of epidemiological data and the medical community’s awareness [2].

Prevalence studies on human Q fever in Brazil were only performed in the southeastern states and Bahia, with prevalence rates ranging from 1.7% to 29% and high rates observed in occupational groups, such as abattoir workers and veterinary students [3–18]. Recent studies in Rio de Janeiro and Minas Gerais, states neighboring São Paulo, showed prevalence rates of 10% and 5.7%, respectively, in the symptomatic population [16,18]. In São Paulo, only two old surveys from the 1950s that investigated *C*. *burnetii* seropositivity in asymptomatic milkers of dairy farms and slaughterhouse workers showed positivity rates of 7% and 1.8%, respectively, using the complement fixation test [3,5]. Some recent reports in the country demonstrate the endemicity of Q fever in the territory, as in an outbreak reported in 2015 in the city of Barbosa-SP (21°160″S 49°5654″W), which affected approximately 16 meatpacking plant workers, and in a family cluster and their companion animals residing in a rural area of Rio de Janeiro [19,20].

In Brazil, studies on the prevalence of animal coxiellosis are scarce; however, in domestic ruminants, Mioni et al. [21] reported 23% (360/1515) seroprevalence in slaughtered cattle from São Paulo state, and Souza et al. [22] reported a seroprevalence of 2.1% (9/403) of sheep and 2.2% (9/412) of goats from Pernambuco state. In São Paulo, reports of abortion episodes in cattle and goats suggest a possible widespread distribution of this bacterium [21,23,24]. Additionally, the presence of *C*. *burnetii* in raw milk samples and cheese from Goiás and Minas Gerais, respectively, sold for human consumption emphasize the public health hazard [25,26]. In addition to ruminants, studies have indicated the possibility of wild reservoirs in the occurrence of the disease. In 2016, the etiologic agent was detected in military firefighters in the region of Ribeirão das Lajes-RJ (22°4128″S43°5149″W) who had contact with goats and capybaras [17]. In 2018, the same group verified the presence of *C*. *burnetii* in bats and wild rodents in the southern states of the country, demonstrating the variety of reservoir possibilities and the need for epidemiological surveys to better characterize the primary reservoirs for human diseases and further implementation of control measures [27,28].

The lipopolysaccharide (LPS) of *C*. *burnetii*, a major virulence factor of this bacterium, can be divided into two phases. Phase I LPS is related to the full-length epitope with the presence of the O-chain. This structure is observed in wild types of *C*. *burnetii* and confers its virulence. Phase II LPS is only observed after several passages in the laboratory using cell culture or other isolation techniques. The phase II LPS is the result of the loss of the O-chain of Phase I LPS related to a gene deletion during laboratory manipulation. *C*. *burnetii* isolates harboring phase II antigens are avirulent and can not be found in nature. Hence, phase II is inserted in the phase I LPS [29–31]. Therefore, acute Q fever immune response to virulent *C*. *burnetii* is initially related to the presence of anti-phase II antibodies, while chronic Q fever is linked to the presence of high titers of anti-phase I antibodies. These data are important for Q fever prevalence studies [29].

*Brucella* spp. is the causative agent of brucellosis, an endemic disease in animals in Brazil, and lacks investigations in human populations. The human incidence of infection follows the incidence of cases in animals; therefore, human brucellosis may be underestimated in São Paulo, considering the status of animal brucellosis in the region with a prevalence of 2.4% [32,33]. In 1953, Amaral et al. [34] conducted a serological survey in the population of São Paulo State and found a positivity rate of 3.5%. In 1991, a serological survey involving professional groups who had contact with positive animals demonstrated a positivity rate of 71% [35]. Although old, these data reinforce the possibility of this disease as a cause of fever of unknown origin in the population of the state nowadays.

Some recent studies on Q fever in Brazil [16,18] used the strategy of sampling patients with suspected dengue fever for serological investigation, and the same approach can be applied for brucellosis, an acute febrile illness. Dengue fever is an endemic disease in Brazil and is suspected in febrile patients with nonspecific symptoms. Clinical signs such as myalgia, headache, and generalized pain, in addition to fever, are present in patients with dengue, Q fever, and brucellosis [36].

Due to the lack of occurrence data of *C*. *burnetii* and *Brucella* spp. in humans in the country, the lack of knowledge of the diseases by health professionals and the range of febrile patients with unknown diagnosis, the aim of this study was to investigate the presence of antibodies against *C*. *burnetii* and *Brucella* spp. infection in patients with systemic clinical signs and negative for dengue, attended by the public health service of the state of São Paulo, Brazil, and therefore contribute to the elucidation of possible differential diagnosis of fever of unknown origin in the state.

## Methods

### Ethics statement

The Research Ethics Committee of Botucatu Medical School–São Paulo State University, Brazil (protocol number 5411/3.720.213) approved this study. Formal consent was waived because it was clarified that medical records were not used, but rather secondary data from coded charts. The data were analyzed anonymously, and the results were presented in an aggregate form, not allowing the identification of the research participants. There was a commitment to respect the precepts of secrecy and confidentiality.

### Sampling

We obtained 604 sera from human patients with symptoms suggestive of dengue, attended by the public health service in 38 municipalities in the state of São Paulo, Brazil. The samples were obtained by convenience and belonged to the serum bank of the Adolfo Lutz Institute in Bauru, São Paulo. The serum bank contained 14,218 serum samples from 2014 to 2017. All sera from the serum bank were tested by ELISA (Dengue IgM Capture ELISA), resulting in 7,062 negative sera for dengue, leaving the diagnosis of their clinical pictures still inconclusive.

A proportional stratification of these negative samples was performed, whose groups were selected by days of symptoms, based on the premise that there was a relationship between the acute illness and the time of symptoms of the patients. Considering that the time required for seroconversion from recent infections is at least seven days, all selected samples from the patients had more than seven days of symptoms. Therefore, extracts were defined as 7–13, 14–20, 21–27, 28–34, 35–41, and > 42 days [37]. A stratification was also made according to the location of the patients, with a proportional amount of patients being collected for each category of municipality. The municipalities of the origin were categorized as rural, small, medium and large, according to the Brazilian Agence Senate classification of 2020 (PLS 316/09).

To define the sample size that would well represent the population of patients with suspected dengue fever, a proportional calculation was performed using open source epidemiological statistics (Openepi). For the calculation, we considered the population of 7,062 individuals negative for dengue with a precision of 3%, 95% confidence interval, and an anticipated frequency of 50% once the prevalence of the disease in the state was unknown [38].

### Serological diagnosis

For *C*. *burnetii*, serodiagnosis was performed using two different immunofluorescence assays described below. Part of the serum samples were tested using an *in-house* assay and part were tested using a commercial assay. In summary, there were enough kits to test 200 samples by the commercial assay, while the other 404 samples needed to be tested with material produced *in-house* by a national reference laboratory. For both assays, the principle of the technique is the same, as well as its methodology, what actually changes is the sensitized antigen on the slides and the possibility of verifying phase II and phase I antigen on commercial slides. For *Brucella* spp. serodiagnosis was performed according to the Plan for the Control and Eradication of Animal Brucellosis and Tuberculosis (PNCEBT), also described below.

#### Q Fever: Indirect immunofluorescence assays (IFA)

The analyses were performed in the Laboratory of Rickettsiosis and Hantaviruses of the Octávio Magalhães Institute, Ezequiel Dias Foundation (Funed-MG). Serology assays used a commercial kit (SCIMEDX Corporation, Denville, New Jersey, USA) containing phase II and phase I antigens of *C*. *burnetii* strain Nine Mile and an *in-house* assay with an antigen produced in embryonated eggs from the Argentinian strain At12 isolated from ticks [39]. Both techniques aimed at detecting total immunoglobulins, but only the commercial test was able to differentiate between phase II and phase I antigens, allowing the characterization of acute and chronic diseases. A portion of the samples (n = 404) were analyzed using the *in-house* technique, and the rest (n = 200) using the commercial kit. The positive and negative controls were from patients previously tested in the laboratory, with positive and negative titers of 1:16.

The serological technique was performed equally for both assays. at 37°C. After another washing and complete drying cycle, the slides were Serum aliquots were diluted 1:64 in phosphate buffered saline (PBS; 0.1M, pH7.2) and deposited on slides containing the antigens (30 μl). The slides were incubated (37°C for 30 min), washed with PBS, and then dried in a humid chamber. Then, 30 μl of fluorescein isothiocyanate (FITC)-conjugated anti-IgM and anti-IgG antibodies were added to the concavities, followed by another incubation in a humid chamber for 30 min mounted with buffered glycerin and coverslip, and the reading was performed under an immunofluorescence microscope (Olympus BX53) with a 40x objective. For each slide used, positive and negative controls of the reaction were prepared in a volume of 30 μl each. The positive samples were serially diluted (1:128, 1:256, 1:512, 1:1024, and so on) until the final titer was reached [39].

#### Brucellosis screening: Rapid serum agglutination with buffered acidified antigen (AAT)

The analyses were performed in the Laboratory of Applied Immunology to Infectious Animal Diseases of the Department of Veterinary Hygiene and Public Health of the São Paulo State University (UNESP) of Botucatu, São Paulo. Serum aliquots (30 μl) and antigen prepared with total *B*. *abortus* cell (sample 1119–3) stained with Rose Bengal (30 μl) were deposited on glass plates and mixed in circular movements for 4 minutes. The agglutination reaction was checked using an indirect light box and the presence of lumps was indicative of a positive reaction. Reactive samples were submitted to confirmatory testing [40].

#### Confirmatory tests for brucellosis: Slow tube serum agglutination (SAL) and 2-mercaptoethanol reaction (2-ME)

The analyses were performed at the Laboratory of Immunology Applied to Infectious Animal Diseases of UNESP, Botucatu, São Paulo. The SAL technique is associated with the 2-ME technique, which is responsible for disrupting disulfide bridges and making a specific detection of IgG present in the serum. Because SAL is sensitive in detecting IgM associated, one technique complements the other [41]. Serum aliquots were diluted in 0.85% saline solution plus 0.5% phenol plus antigen (SAL solution) and in 0.1M 2-mercaptoethanol solution without phenol plus antigen (2-ME solution) at concentrations of 1:25, 1:50, 1:100 and 1:200. After shaking well, the tubes were incubated (37°C for 48 h). The reading of the reactions were done with the help of an indirect light source in a dark-bottomed box. With gentle agitation of the tubes, when a translucent supernatant was observed and the lumps were not broken, the reaction was considered positive [40].

### Data analysis

The distribution of continuous variables was assessed using normality tests (Shapiro-Wilk) and graphical analyses (QQ plot and histogram). A generalizied linear model of logistic regression was used to calculate the association between Q fever positivity and patients’ symptom duration, age, sex, and type of city of residence. After verifying no interaction between each other variabels were present, one variable at a time was evaluated and odds ratio were reported. Non-parametric tests were used to determine differences between median ages with positive and negative sera (Wilcoxon) and median days with fever associated with positives and negatives (Wilcoxon). Statistical associations were considered when p-value was <0.05. The analyses were performed using Statistical Analysis Software (SAS) (SAS Institute, USA).

## Results

**Fig 1:**
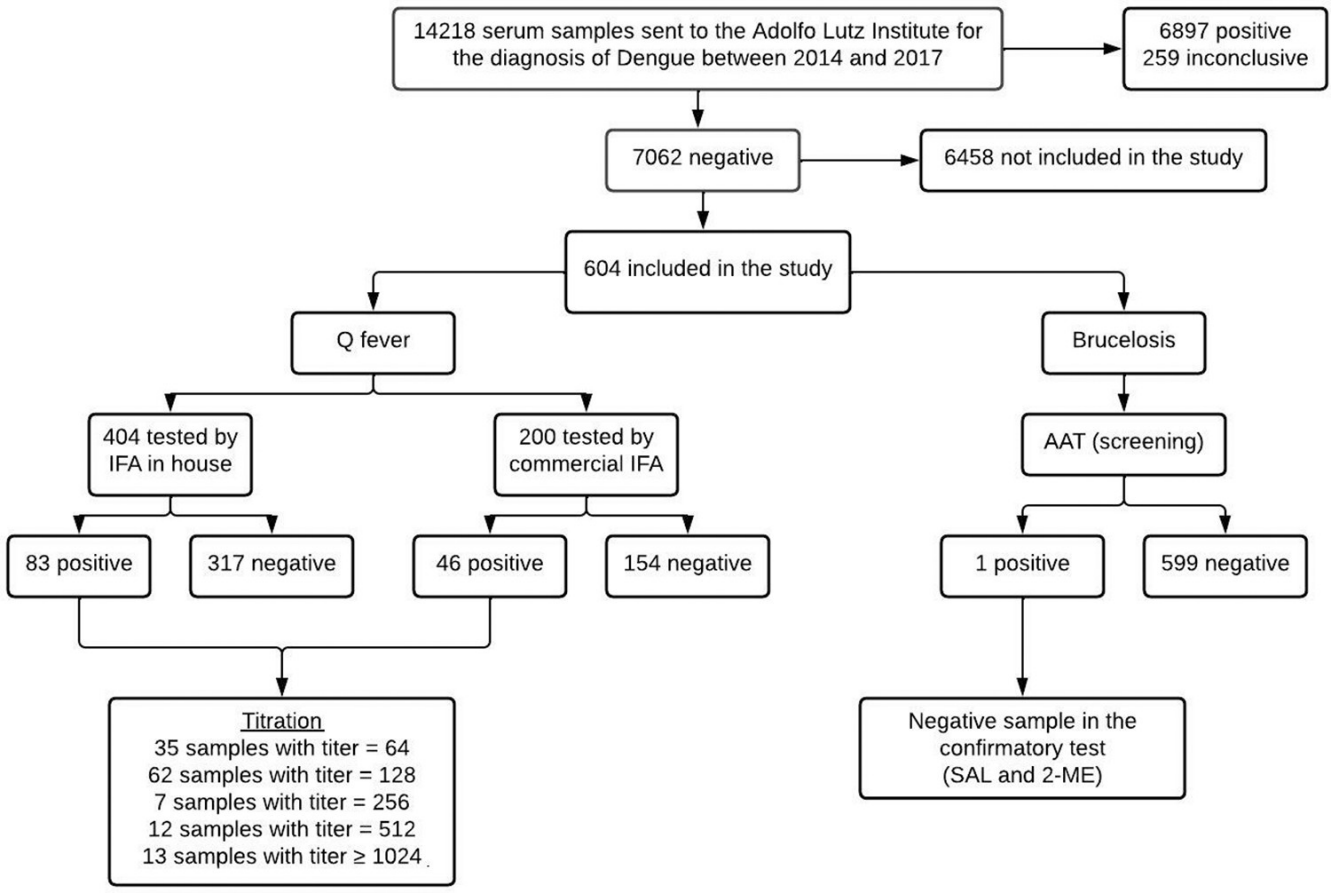
Flowchart of the samples and analysis results of the study.

Fig 1 shows the flowchart of the study design, from the origin of the samples, through screening, to the confirmatory tests.

All samples tested were negative for brucellosis. One patient was reactive to the disease through AAT. However, the individual was classified as negative in the confirmatory test, discarding the disease status.

Seropositivity of antibodies against *C*. *burnetii* was observed in 129 samples (21.36%) (95% CI 18.15–24.85), revealing past exposure to the pathogen. Among the positive samples, 91 patients had IgM antibodies (70.5%) and 111 had IgG antibodies (86.0%). Most samples (56.6%) were positive for both antibodies, 14% were positive for IgM, and 29.5% were positive for IgG. Regarding the antibodies phase, 42 patients had anti-phase II antibodies (91.3%), and 16 had anti-phase I antibodies (34.8%). Most samples (65.2%) were positive for phase II, 8.7% were positive for phase I, and 26.1% were positive for both antibodies. These results can be seen in Tables 1 and 2.

**Table 1:** Detection of anti-*C*. *burnetii* IgM and IgG antibodies.

IFA (n = 604)					
Serology positive	IgM antibodies	IgG antibodies	IgM/IgG		
			+/+	+/-	-/+
129 (21.3%)	91 (70.5%)	111 (86.0%)	73 (56.6%)	18 (14.0%)	38 (29.5%)

IFA: in-house and commercial indirect immunofluorescence assay

Data are expressed as n % or n.

Serology positive: number of positives concerning the 604 samples

IgM and IgG antibodies: type of antibody detected concerning positive +/+:IgM and IgG positive

+/-: IgM positive and IgG negative

-/+: IgM negative and IgG positive

**Table 2:** Detection of anti-*C*. *burnetii* phase II and phase I antibodies.

IFA commercial (n = 200)					
Serology positive	Reaction ag/ab phase II	Reaction ag/ab phase I	phase II/phase I		
			+/+	+/-	-/+
46 (23%)	42 (91.3%)	16 (34.8%)	12 (26.1%)	30 (65.2%)	4 (8.7%)

IFA commercial: commercial indirect immunofluorescence assay

Data are expressed as n % or n.

Serology positive: number of positives concerning the 200 samples

Reaction ag/ab phase II and phase I: type of antibody detected concerning positives

+/+: phase II and phase I positive

+/-: phase II positive and phase I negative

-/+: phase II negative and phase I positive

Titration of positives was performed to establish whether past exposure was a characteristic of the disease. According to Anderson et al. [29], a cut-off of 64, 128, and 1024 was set for past exposure (seropositivity), acute disease, and chronic disease, respectively, together with the presence of anti-phase II and anti-phase I antibodies in the sera. The presence of anti-phase II together with a cut-off of 128 was correlated with acute disease, and the presence of anti-phase I with a cut-off of 1024 or higher was considered a chronic disease. The results are shown in Table 3.

**Table 3:** Distribution of Q fever seropositivity according to the titer and type of antibody (ab).

Titles	ab phase II	ab phase I
= 64	12	4
= 128	24	8
= 256	2	1
= 512	3	1
≥1024	0	3
	acute	chronic

First highlight: patients with titers and antibodies indicative of acute disease

Second highlight: patients with titers and antibodies indicative of chronic disease

Of the 46 seropositive patients by commercial immunofluorescence test, 29 (63%) had phase II antibody titers higher than 128 and were therefore defined as having acute Q fever. Regarding chronic Q fever, three patients (6.5%) had phase I antibody titers higher than 1024, suggesting that these patients had a persistent infection. A 60-year-old woman, symptomatic for more than 14 days, had an extremely high titer of 32.768 for both phases.

Table 4 presents the results of the serological analyses according to the variables collected: days of symptoms, age, sex, and size of the municipalities classified as rural, small, medium, and large. The table shows the seropositive and seronegative patients, total population, and percentages, allowing an in-depth analysis of these groups described in the literature as more or less likely to acquire the disease.

**Table 4:** Clinical and sociodemographic characteristics of febrile patients in São Paulo state, Brazil (2014–2017) and their respective seropositivities for Q fever.

	*C*. *burnetii* seronegative		*C*. *burnetii* seropositive		Total population
Variables	N	%	N	%	n
Symptom Time					
7–13 days	334	82.9	69	17.1	403
14–20 days	89	69.5	39	30.5	128
21–27 days	24	64.9	13	35.1	37
28–34 days	9	81.8	2	18.2	11
35–41 days	10	71.4	4	28.6	14
≥ 42 days	9	81.8	2	18.2	11
Age					
Young	127	81.4	29	18.6	156
Adults	287	76.9	86	23.1	373
Seniors	61	81.3	14	18.7	75
Sex					
Women	257	80.3	63	19.7	320
Men	218	76.8	66	23.2	284
Size of city					
Large	192	79.3	50	20.7	242
Medium	53	86.9	8	13.1	61
Small	171	78.4	47	21.6	218
Rural	59	71.1	24	28.9	83

Most patients were adults with 7 to 13 days of symptoms. However, when comparing seropositivity to the total samples for each symptom duration, it was observed to be highest in patients with 14–20 and 21–27 days of symptoms. Concerning sex, positive results were similar between the groups. Regarding the type of city, most patients were from large cities, but in contrast, populations from rural municipalities showed higher positivity than the other populations.

Logistic regression was performed to determine whether there was a statistically significant association between the groups. Regarding the duration of symptoms, patients with 14–20 days of symptoms had 2.12 (95% CI 1.34–3.35) times more chances of being seropositive for Q fever than patients with 7–13 days, and patients with 21–27 days of fever had 2.62 (95% CI 1.27–5.41) times more chances of being seropositive for Q fever than patients with 7–13 days. For the other variables analyzed, there were no significant associations between the groups. These and other results from the logistic regression can be seen in Table 5.

**Table 5:** Odds Ratio and 95% Confidence Interval for *C*. *burnetii* seropositivity by patients´ symptom duration, categorized age, sex and type of city of residence.

Variable		Odds Ratio	Lower 95% CI*	Upper 95% CI*	Statistical Difference (p<0,05)
Symptom Time					
21–27	14–20	1.24	0.57	2.68	No
14–20	28–34	1.97	0.41	9.58	No
14–20	35–41	1.10	0.32	3.72	No
**14–20**	**7–13**	**2.12**	**1.34**	**3.35**	**Yes**
14–20	> = 42	1.97	0.41	9.58	No
21–27	28–34	2.44	0.46	13.05	No
21–27	35–41	1.35	0.35	5.19	No
**21–27**	**7–13**	**2.62**	**1.27**	**5.41**	**Yes**
21–27	> = 42	2.44	0.46	13.05	No
35–41	28–34	1.80	0.26	12.34	No
28–34	7–13	1.08	0.23	5.10	No
28–34	> = 42	1.00	0.11	8.77	No
35–41	7–13	1.94	0.59	6.37	No
35–41	> = 42	1.80	0.26	12.34	No
> = 42	7–13	1.08	0.23	5.10	No
Age					
Adult	Seniors	1.31	0.70	2.45	No
Adult	Young	1.31	0.82	2.10	No
Seniors	Young	1.01	0.49	2.04	No
Sex					
Male	Female	1.24	0.84	1.82	No
Size of city					
Large	Medium	1.73	0.77	3.87	No
Small	Large	1.06	0.67	1.65	No
Rural	Large	1.56	0.88	2.76	No
Small	Medium	1.82	0.81	4.10	No
Rural	Medium	2.69	0.99	6.52	No
Rural	Small	1.48	0.83	2.63	No

*CI—Confidence Interval

**Fig 2:**
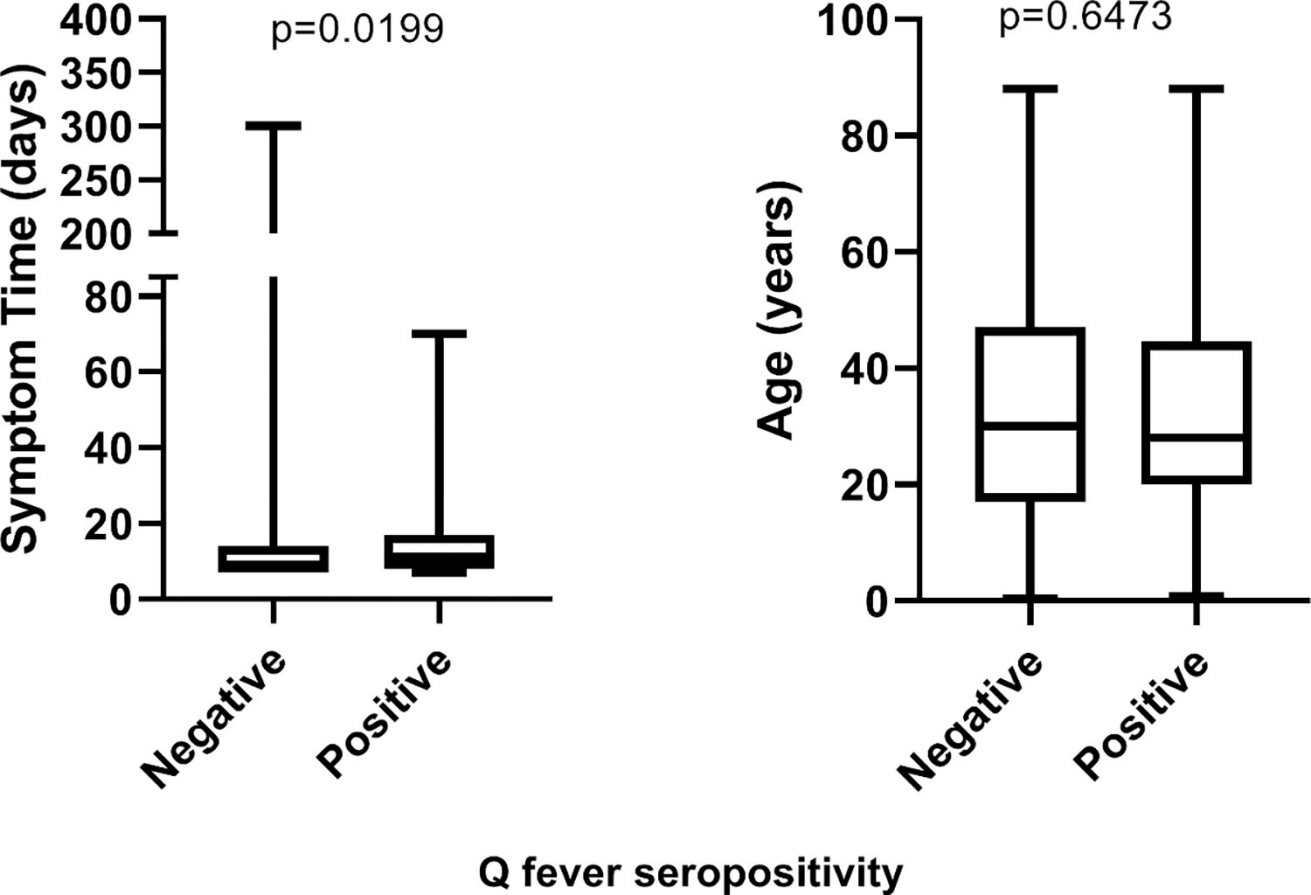
Distribution of symptom time (days) and age (years) by positivity for Q fever. P-values lower than 0.05 were considered statistically different.

Additionally, for the non-parametric data, the medians of the positives and negatives were established, allowing significant associations to be observed with respect to Q fever. The results are presented in Fig 2.

In the first chart, we see that the median (Min-Max) of positives regarding symptom duration was 11 (7–70) days, while the median of negatives was 9 (7–300) days. A significant difference was observed between the medians, indicating an association between symptom duration and Q fever seropositivity. In the second chart, we see that the median of positives in relation to age was 30 (0.3–88) years old, while the median of negatives was 28 (0.75–88) years old. In contrast, no statistically significant association was observed between the medians with respect to patient age.

## Discussion

The IFA showed an expressive positivity of 21.4% (129/604) for Q fever, in contrast to brucellosis serology, indicating that *C*. *burnetii* may play a more critical role in febrile illness in the state of São Paulo than smooth brucellae.

Based on the ability of *C*. *burnetii* to travel long distances in the form of spore-like structures and the fact that ruminants eliminate the agent at the time of birth or abortion, we believe that the presence of animal herds in the region may explain the high seropositivity of the urban and rural population of the municipalities [42,43]. On the other hand, Brucellosis is probably not a differential diagnosis for Dengue-like patients.

Most patients had titers of 128 at IFA, which was expected for patients with clinical symptoms. Being aware that 63% of the positive patients analyzed had acute Q fever, the hypothesis that some of the patients sent for dengue diagnosis could be sick because of Q fever is reinforced. It is essential to be conscious of the risk of these positive acute patients in developing a persistent infection leading to more severe clinical outcomes in the future that can affect vital organs such as the heart and central nervous system, and the development of serious problems such as pneumonia and granulomatous hepatitis [44]. Chronic disease becomes more challenging to treat once the bacteria adopt more sophisticated survival mechanisms and persist in protected organs where the action of antibiotics is hindered. This is a difficulty encountered in patients defined as chronic in the study and who are at greater risk of developing the complications of the disease [42].

The association between symptom duration and Q fever seropositivity was supported by statistical analysis. According to Anderson et al. [29], 90% of patients seroconvert in the third week of illness. This percentage could not be assessed in our study because we selected a specific number of patients at different times of symptoms. However, from logistic regression, we noted that patients with 21–27 days of symptoms were 2.62 times more positive than patients with 7–13 days, and a similar result occurred in patients with 14–20 days, who were shown to be 2.12 times more positive than patients with 7–13 days of symptoms. The importance of paired serology of patients, which occurs more frequently in the diagnosis of other infectious diseases, is reinforced here. To this end, two serum samples collected at 7–21 day or 7–28 day intervals in order to observe the increase in antibody titer could ensure a more accurate diagnosis of Q fever. Furthermore, these results demonstrate that Q fever is a diagnostic possibility for febrile patients with more than two weeks of symptoms, and a disease to be considered by the brazilian medical community to reduce underdiagnosis.

The number of infected patients concerning age was well distributed, and the statistics did not point to any associations, although the majority (30%) were between 20 and 29 years of age. According to Anderson et al. [29], the older the patient, the greater the chance of having Q fever, which was not observed in our study. One result that should be carefully considered was that two of the positive patients were less than 1 year old. Milazzo et al. [45] reported that newborn children could become infected due to mother-to-child descent transmission through breastfeeding or as a result of hospital infection.

The association between positivity and sex was also not demonstrated. Both sexes appeared equally susceptible, although men were slightly more affected.

Considering that this disease affects a broad range of animal species in Brazil [21–24,28] and that domestic ruminants are the main reservoir of human infection [46,47], it was hypothesized that people from exclusively rural municipalities would have higher seropositivity results than those from municipalities with large urban centers. However, the airborne transmission of *C*. *burnetii* may explain the lack of association between the types of municipalities in the study and Q fever positivity. The presence of livestock in areas surrounding cities can threaten public health in the presence of a high prevalence of the disease in animals and shedding by asymptomatic herds during the breeding season, leading to environmental contamination [43].

Initially, we intended to use the commercial immunofluorescence kit to analyze all samples, considering that the commercial kit contemplates analyzing antibody phases present in the serum. However, the availability of these kits in Brazil is scarce, and there is limited importation of these diagnostic materials by national reference laboratories. Despite this difficulty, the samples analyzed with the commercial kit allowed us to observe the presence of antibodies anti-phase I and II in some of these patients and the presence of antibody titers related to acute and chronic Q fever. Furthermore, *in-house* immunofluorescence proved to be efficient in seropositivity analysis, although there is a need for a study comparing techniques with the results obtained to confirm this efficiency.

It is essential to perform studies involving molecular detection of *C*. *burnetii* in the early phase of the disease (10 days or less of symptoms), which would characterize an active infection before seroconversion with an exact time of disease occurrence and past exposure to the agent [44]. In addition, as with most studies involving samples from a serum bank, the epidemiological data from patients are not complete, and studies with broader epidemiological questionnaires are needed to cover all possibilities of the source of animal infection and associated risk factors. This result does not include the general population of the state of São Paulo, considering that these are patients with clinical symptoms and approximately 60% of infected people are usually asymptomatic. These data indicate that there may be more seropositive people than we know because of the presence of asymptomatic cases [48].

This is an important study given the fact that it assesses seropositivity in patients with pyrexia of unknown origin, who do not necessarily have contact with animals or laboratories, and because it is an investigation in a country that is unfamiliar with Q fever and lacks investigative studies. Our results related to the distribution of seropositive cases throughout the state can be used as a reference to guide surveillance and prevention activities and other epidemiological studies that may come into existence. Q fever is a diagnostic possibility for patients with dengue-like symptoms and is a cause for attention by Brazilian health surveillance agencies.

## Conclusions

This study showed seropositivity for *C*. *burnetii* antibodies in 21.4% of the sampled patients with a negative diagnosis of dengue fever. The titers evaluated and types of antibodies present suggest the occurrence of Q fever in its acute and chronic forms. Statistical associations were observed between positive serology for Q fever and duration of symptoms. The absence of seropositivity for *Brucella* spp. antibodies has been demonstrated. In this study, Q fever had a higher seropositivity rate than brucellosis, indicating that the disease may play a more critical role in febrile illness in the central-western region of the state of São Paulo. Thus, healthcare professionals should be aware of the existence of the disease and consider *C*. *burnetii* in the differential diagnosis of influenza-like symptoms. Our results related to the distribution of seropositive cases throughout the state can be used as a reference to guide surveillance and prevention activities and other epidemiological studies that may come into existence. Q fever is a diagnostic possibility for patients with dengue-like symptoms and is a cause for attention by Brazilian health surveillance agencies.
